# Justice under tourism pressure: Governance fairness, emotional solidarity, and residents’ co-governance participation intention in tourism-affected communities

**DOI:** 10.1371/journal.pone.0360394

**Published:** 2026-10-12

**Authors:** Xiaoqing Jiang

**Affiliations:** Department of Tourism Management, School of Business, Wuxi Taihu University, Wuxi, China; I-Shou University, TAIWAN

## Abstract

Amid growing tourism pressure in communities surrounding major attractions, questions of governance legitimacy, fairness, and resident engagement have become increasingly salient. This study examines how residents’ perceptions of distributive, procedural, and interactional justice are associated with emotional solidarity with tourists and co-governance participation intention. Survey data from 308 residents living near major attractions in Wuxi, China, were analyzed using structural equation modeling, mediation analysis, and hierarchical regression analysis. The results show that all three dimensions of perceived justice are positively associated with emotional solidarity and co-governance participation intention, with procedural and interactional justice showing particularly strong associations. The mediation results indicate a significant indirect association between governance fairness and participation intention through emotional solidarity. The moderation results show that perceived tourism pressure is significant only for the association between interactional justice and emotional solidarity, while its moderating role is not significant for distributive or procedural justice. These findings suggest that residents’ willingness to engage in co-governance is associated not only with how tourism is managed institutionally, but also with their relational orientation toward tourists. The study highlights the importance of transparent procedures, meaningful resident voice, and respectful everyday interactions for understanding collaborative governance in urban destinations under sustained tourism pressure.

## 1. Introduction

Tourism-affected communities are increasingly confronted with a governance paradox: tourism is promoted as an engine of economic vitality, yet sustained visitor intensity is often associated with pressures on everyday liveability, distributional conflicts, and legitimacy disputes over how tourism is managed [1,2]. In such contexts, tourism pressure is important not only as a matter of visitor density or carrying capacity, but also as a governance condition under which questions of fairness, voice, accountability, and public tolerance become more visible [3]. Empirical studies show that rapid tourism expansion is associated with pressures on housing, public space, and community cohesion, prompting residents to evaluate tourism development through a fairness lens rather than through economic performance alone [4,5]. These dynamics suggest that resident–tourism relations in high-intensity settings are increasingly linked to how governance is experienced in everyday life—who gains and who loses, who is heard, and whether authorities engage residents with transparency and respect [6,7].

Existing research has commonly explained residents’ tourism responses through social exchange theory, which emphasizes perceived benefits, costs, reciprocity, and balanced exchange [8–10]. This perspective has been useful for understanding residents’ attitudes and support for tourism development [11,12]. In parallel, emotional solidarity theory highlights the affective bonds between residents and tourists, including perceived closeness, mutual understanding, and emotional connection, and has been linked to residents’ tourism evaluations and behavioral orientations [13,14]. Recent studies further suggest that emotional solidarity is associated with interactional dynamics, contextual shocks, stereotypes, environmentally responsible behavior, and other pro-social outcomes [15–18]. Together, these perspectives suggest that residents’ responses under high-intensity tourism are embedded not only in evaluations of tourism impacts, but also in institutional judgments and host–guest relational orientations.

Despite these advances, three important gaps remain. First, although perceived justice theory is well developed in organizational and social psychological research, tourism scholarship has often treated fairness implicitly, embedding it within general impact or exchange assessments rather than explicitly modeling it as a multidimensional governance judgment [19–21]. Justice theory distinguishes distributive justice, procedural justice, and interactional justice, and these dimensions may be differently associated with how residents evaluate tourism governance [22,23]. In tourism settings marked by sustained visitor pressure, such distinctions are especially important because residents evaluate not only tourism outcomes, but also how tourism-related decisions are made, communicated, and implemented in everyday governance practice.

Second, while emotional solidarity has been widely examined as an outcome of host–guest interaction and as a predictor of residents’ support for tourism, its role as a relational mechanism linking governance evaluations to participatory intentions remains underexplored [13,15,24,25]. Participatory governance requires residents not only to accept tourism development, but also to engage in dialogue, monitoring, problem reporting, and other forms of co-governance. If tourism governance fairness is associated not only with how residents evaluate authorities but also with how they feel toward tourists, emotional solidarity may serve as a socio-emotional pathway linking governance legitimacy to co-governance participation intention.

Third, although participatory and “good governance” models are increasingly promoted in tourism, empirical evidence remains limited regarding the conditions under which residents report stronger intention to participate in co-governance in settings marked by sustained tourism pressure [26,27]. Relatedly, tourism intensity, stress, and pressure have been associated with residents’ emotions and behavioral intentions, yet it remains unclear whether perceived tourism pressure conditions the association between justice judgments and socio-emotional ties [28–31]. Addressing this boundary issue can help clarify whether fairness operates as a relatively stable interpretive framework or whether its relational implications vary across different pressure conditions.

To examine these issues empirically, this study is situated in Wuxi, a major tourism city in the Yangtze River Delta that hosts multiple national 5A-level attractions and receives sustained high volumes of domestic visitors. Official statistics indicate that Wuxi welcomed 146.46 million domestic tourist visits in 2024 and generated CNY 175.21 billion in tourism revenue [32]. Peak-season pressures are also pronounced: during the 2025 Spring Festival holiday alone, the city received 15.56 million visitors [33]. These high and volatile flows make governance processes, institutional responsiveness, and routine interactional practices highly visible to surrounding communities, providing a relevant setting for investigating justice-based governance evaluations under sustained tourism pressure.

Building on perceived justice theory, social exchange theory, and emotional solidarity research, this study develops an integrative framework linking residents’ perceptions of distributive, procedural, and interactional justice to emotional solidarity with tourists and intention to participate in tourism co-governance, while considering perceived tourism pressure as a contextual boundary condition. Accordingly, this study addresses three research questions: (RQ1) How are different dimensions of perceived justice in tourism governance associated with residents’ emotional solidarity with tourists? (RQ2) How are tourism governance fairness perceptions and emotional solidarity associated with residents’ intention to participate in co-governance, and is there an indirect association through emotional solidarity? (RQ3) Does perceived tourism pressure moderate the association between tourism governance fairness and emotional solidarity?

This study contributes to tourism governance research in three ways. First, it explicitly models distributive, procedural, and interactional justice as complementary yet distinct governance evaluations, thereby extending justice-based explanations of resident responses in tourism-affected communities. Second, it shifts attention from residents’ general support for tourism development to their intention to participate in tourism co-governance, highlighting a more active and governance-oriented form of resident engagement. Third, it examines emotional solidarity as a relational mechanism linking governance fairness to co-governance participation intention and considers perceived tourism pressure as a contextual condition. Practically, the findings provide guidance for destinations facing sustained tourism pressure by highlighting the importance of transparent procedures, meaningful resident voice, and respectful everyday interactions for supporting collaborative destination management.

## 2. Literature review

### 2.1. Perceived justice theory

Justice has long been a central concern in social and political theory, providing a normative basis for assessing the legitimacy of institutional arrangements and governance outcomes. Rawls’ [34] concept of justice as fairness established a foundational principle for evaluating whether social systems protect equal basic liberties and structure opportunities in ways that do not disadvantage vulnerable groups. Building on this normative tradition, social psychology and organizational research shifted attention from abstract principles to how individuals perceive and evaluate fairness in everyday institutional interactions, giving rise to perceived justice theory [20,21]. This perspective emphasizes that attitudes and behaviors are associated not only with objective outcomes but also with subjective assessments of whether outcomes, decision-making processes, and interpersonal treatment are considered fair. Accordingly, perceived justice is commonly conceptualized as a multidimensional construct comprising distributive justice, procedural justice, and interactional justice. Distributive justice concerns the fairness of benefit–burden allocation, procedural justice concerns the fairness of decision rules, including consistency, transparency, and voice, and interactional justice concerns respectful treatment and adequate communication [19,22]. Together, these dimensions provide a comprehensive analytical framework for examining how individuals assess institutional legitimacy and respond to governance arrangements across diverse social contexts.

The relevance of perceived justice becomes particularly salient under conditions of overtourism, which is increasingly conceptualized not merely as excessive visitor density but as a governance challenge associated with multiple forms of injustice in residents’ everyday lives [3,35]. Empirical studies show that rapid tourism growth is often associated with intensified housing pressures, restricted access to public space, and weakened social cohesion, prompting residents to evaluate tourism development through a fairness lens rather than solely in terms of economic performance [4,5]. These evaluations often involve distributive concerns over how tourism benefits and burdens are shared, procedural concerns regarding residents’ inclusion in decision-making, and interactional concerns related to respect, transparency, and communication in governance practices [6,7]. Recent justice-oriented tourism research further argues that effective responses to overtourism depend not only on technical visitor-management tools, but also on whether governance arrangements are locally grounded, inclusive, and responsive to community needs [27,36]. Consistent with this view, Gorji et al. [2] show that residents often interpret overtourism pressures through a justice lens, attributing everyday strains to unfair governance and linking perceived injustice to heightened social tension and demands for more equitable urban futures. Together, this body of work positions perceived justice as a key analytical lens for understanding residents’ responses to tourism governance in high-pressure destinations.

Within this theoretical context, perceived justice offers a particularly appropriate lens for examining tourism governance under conditions of overtourism. Rather than treating residents’ reactions as direct responses to objective tourism impacts, perceived justice theory emphasizes how governance arrangements are evaluated through everyday experiences of benefit allocation, decision-making procedures, and interpersonal treatment [23,37]. Recent tourism research suggests that residents’ interpretations of congestion, housing pressure, and the loss of public space are often framed not simply as unavoidable consequences of tourism growth, but as reflections of whether governance processes are fair, inclusive, and respectful [2,6,7]. In this sense, distributive, procedural, and interactional justice constitute analytically distinct yet interrelated dimensions through which residents make sense of tourism governance outcomes in high-pressure destinations. Focusing on these justice perceptions allows tourism governance to be examined as a legitimacy-related institutional process, rather than solely as a technical or managerial response to overtourism. Moreover, such justice perceptions may be associated not only with residents’ evaluations of governance institutions, but also with how they attribute tourism-related pressures and interpret the social presence of tourists in everyday community life.

### 2.2. Social exchange theory

Social exchange theory (SET) explains how individuals form attitudes and behavioral intentions by evaluating reciprocal benefits and costs in social relationships. Rooted in classical sociology and social psychology [38], contemporary SET highlights that support for collective arrangements is more likely when exchanges are perceived as mutually beneficial, fair, and sustainable [10]. Exchanges also involve relational and emotional returns, including trust, respect, and recognition, which are associated with sustained cooperation over time [11].

In tourism research, SET has been widely used to understand residents’ responses to tourism development and governance. Residents tend to support tourism when perceived benefits outweigh social, environmental, and cultural costs [8,9]. Under overtourism, such evaluations become more governance-sensitive: residents increasingly interpret outcomes as exchanges with both institutions and tourists, linking reciprocity and fairness to acceptance and support [2,12].

SET also provides a bridge between governance perceptions and resident–tourist relationships. Respectful interaction and shared benefits may be associated with stronger emotional solidarity, whereas imbalanced exchanges may be associated with lower trust and wider social distance [13,39]. Empirical evidence shows that resident–tourist interaction is positively associated with destination immersion and relational outcomes [40], and that community attachment and emotional solidarity operate as exchange-based mechanisms linked to support for sustainable tourism [24]. Overall, SET supports the expectation that perceived fairness in tourism governance is associated with stronger emotional solidarity and higher residents’ co-governance participation intention under overtourism pressures.

### 2.3. Research hypotheses development

#### 2.3.1. The relationship between perceived justice and emotional solidarity.

In tourism governance contexts, residents’ fairness evaluations are consequential because they are associated with how residents interpret tourism as a legitimate or illegitimate social arrangement [21]. Perceived justice, typically conceptualized through distributive, procedural, and interactional dimensions, captures whether residents view tourism benefits and burdens as appropriately allocated, decision processes as inclusive, and governance interactions as respectful and communicative [1,22]. Empirical evidence shows that these justice perceptions are closely tied to residents’ broader tourism-related responses, including quality of life appraisals and support for tourism development, indicating that fairness operates as a key psychological lens through which residents make sense of tourism governance outcomes [23,41].

From a social exchange perspective, perceived justice functions as a diagnostic cue of exchange balance and reciprocity in the tourism system. It is important to note that perceived justice and emotional solidarity are directed toward different referents: the former concerns residents’ evaluations of governance institutions and processes, whereas the latter concerns residents’ affective orientation toward tourists. When governance is perceived as fair, residents are more likely to infer that tourism is managed in a way that recognizes community contributions, addresses undue burdens, and maintains a more balanced resident–tourism exchange [10]. Such evaluations may be associated with trust in authorities and perceived governance legitimacy, but their implications are not limited to institutional judgments. In tourism-affected communities, residents encounter tourists within a governance environment that regulates visitor flows, public-space use, communication, compensation, and conflict management [1]. Thus, governance fairness may also be linked to how residents attribute tourism-related pressures and interpret tourists’ presence in everyday community life.

This spillover mechanism is especially relevant under overtourism conditions. When governance is perceived as unfair, residents may interpret congestion, crowding, and competition for public space as signs that tourism privileges visitors over local needs, making tourists more likely to be viewed as the visible source of everyday disruption. By contrast, when governance is perceived as fair, inclusive, and responsive, residents have stronger grounds to interpret tourists’ presence as legitimate and less threatening [41]. In this sense, institutional justice may extend beyond evaluations of authorities by informing the relational meaning residents attach to tourists.

Emotional solidarity represents residents’ overall affective bond with tourists, reflecting perceived closeness, mutual understanding, and positive emotional connection in host–guest relations [13]. When residents interpret tourists’ presence within a fair and reciprocal governance context, they may be more likely to report emotional connection rather than social distance [42]. Consistent with this reasoning, recent work suggests that justice perceptions and emotional solidarity are intertwined in residents’ tourism-related evaluations, and positive host–tourist interactions are associated with stronger emotional solidarity and lower negative social categorization [16,37]. Accordingly, when tourism governance is perceived as fair, residents are more likely to report positive emotional bonds with tourists and stronger resident–tourist relationship quality. Therefore, the following hypothesis is proposed:

***H1: Residents’ perceived justice in tourism governance is positively associated with emotional solidarity with tourists.***

***H1-1: Distributive justice is positively associated with emotional solidarity with tourists.***

***H1-2: Procedural justice is positively associated with emotional solidarity with tourists.***

***H1-3: Interactional justice is positively associated with emotional solidarity with tourists.***

#### 2.3.2. The relationship between perceived justice and intention to participate in co-governance.

Co-governance participation intention refers to residents’ stated willingness to contribute to tourism-governance processes through participatory channels such as consultation meetings, problem reporting, suggestion provision, and volunteer activities related to destination management. In the context of overtourism, such participation reflects residents’ intended involvement in the collective management of tourism-related pressures, public-space use, and community interests. This construct therefore captures residents’ readiness to provide voice, local knowledge, feedback, and practical support for destination governance, while referring to intended participation rather than observed co-governance behavior or general pro-social destination behavior [22,43].

Perceived justice in tourism governance is expected to be positively associated with residents’ intention to participate in co-governance. From a social exchange perspective, distributive, procedural, and interactional fairness can be interpreted as signals of reciprocity and legitimacy, which may be associated with residents’ willingness to reciprocate through constructive engagement rather than withdrawal [10,44]. In tourism settings, governmental fairness has been shown to be associated with residents’ reactions to tourism development and their supportive orientations toward destination management [22,45]. This association may be particularly salient under overtourism, where pressures on public space and common resources make residents more attentive to whether governance arrangements protect community interests and provide meaningful voice. Participatory governance is therefore viewed not only as an institutional arrangement, but also as a corrective mechanism for addressing perceived spatial and distributive imbalances in high-pressure destinations [2,26]. Recent evidence further indicates that justice perceptions are intertwined with residents’ broader tourism-related evaluations and pro-destination responses, implying that fairness may provide a motivational basis for deeper involvement in governance processes [37]. Therefore, residents who perceive tourism governance as fair are more likely to report stronger intention to participate in co-governance. Accordingly, we propose the following hypothesis:

***H2: Residents’ perceived justice in tourism governance is positively associated with their intention to participate in co-governance.***

***H2-1: Distributive justice is positively associated with residents’ intention to participate in co-governance.***

***H2-2: Procedural justice is positively associated with residents’ intention to participate in co-governance.***

***H2-3: Interactional justice is positively associated with residents’ intention to participate in co-governance.***

#### 2.3.3. The relationship between emotional solidarity and co-governance participation intention.

Emotional solidarity captures residents’ affective bond with tourists, reflected in perceived closeness, mutual understanding, and a sense of connection in host–guest relations [46]. Such relational affinity matters because it is associated with lower social distance and a greater tendency to view tourists as interaction partners rather than outsiders, thereby corresponding to lower defensiveness and greater willingness to engage in collective destination stewardship. Recent evidence shows that emotional solidarity is associated with reduced stereotypes and negative social categorization, as well as more constructive orientations toward tourists [16], and that it is responsive to contextual shocks, being linked to how residents re-evaluate host–guest ties and destination-related behaviors [17]. Moreover, emotional solidarity has been linked to a range of pro-destination and pro-social outcomes, including support for tourism development and environmentally responsible behavior, suggesting that affective connection may be associated with action-oriented engagement [15,18].

In the context of tourism co-governance, this action-oriented engagement is understood as residents’ willingness to contribute to governance-related processes rather than as general pro-social destination behavior. Residents who feel emotionally connected to tourists may be more inclined to provide feedback, offer suggestions, and support community-based destination management because they are more likely to view host–guest relations as worth maintaining and improving [2]. Importantly, recent work indicates that emotional solidarity operates as a key psychological mechanism through which residents’ community commitment and concern are linked to supportive behaviors [25]. Extending this logic to participatory governance, residents who feel emotionally connected to tourists are more likely to report stronger intention to participate in tourism co-governance processes. Accordingly, we propose the following hypothesis:

***H3: Residents’ emotional solidarity with tourists is positively associated with their intention to participate in co-governance.***

#### 2.3.4. The mediating role of emotional solidarity in the relationship between perceived justice and co-governance participation intention.

Perceived justice in tourism governance may be associated with residents’ co-governance participation intention not only through direct motivational pathways, but also indirectly through residents’ relational orientation toward tourists. From a social exchange perspective, fair governance arrangements signal reciprocity and respect, which may be associated with stronger emotional solidarity by corresponding to lower social distance and higher perceived mutual understanding in host–guest relations [47,48]. Emotional solidarity, in turn, represents a socio-emotional mechanism through which positive relational evaluations may be linked to stronger intended engagement.

Prior studies indicate that emotional solidarity mediates the associations between antecedent perceptions, such as sincerity, authenticity, and relational cues, and support-related or responsible behavioral intentions [49,50]. Moreover, recent evidence highlights the close interplay between fairness-related appraisals and emotional solidarity in relation to behavioral intentions, suggesting a plausible indirect pathway from governance perceptions to participatory responses [41]. Accordingly, emotional solidarity is likely to mediate the association between perceived justice in tourism governance and residents’ co-governance participation intention. Therefore, we propose the following hypothesis:

***H4: Emotional solidarity mediates the association between residents’ perceived justice in tourism governance and their co-governance participation intention.***

***H4-1: Emotional solidarity mediates the association between distributive justice and co-governance participation intention.***

***H4-2: Emotional solidarity mediates the association between procedural justice and co-governance participation intention.***

***H4-3: Emotional solidarity mediates the association between interactional justice and co-governance participation intention.***

#### 2.3.5. The moderating role of residents’ perceived tourism pressure in the association between perceived justice and emotional solidarity.

Residents’ perceived tourism pressure refers to residents’ subjective appraisal of tourism-related demands, disruptions, and stressors in daily life [28,29]. In high-pressure tourism settings, residents’ socio-emotional orientations toward tourists may become more sensitive to contextual strain, making the association between governance fairness and host–guest affective bonds more contingent on perceived pressure. Empirical evidence indicates that tourism-related stress is associated with residents’ emotional solidarity and quality-of-life outcomes [30], while broader tourism intensity is linked to residents’ behavioral intentions and pro-tourism responses [31]. Stress- and pressure-related appraisals also help explain variation in residents’ support-related judgments toward tourism development [51]. Taken together, these findings suggest that residents’ perceived tourism pressure may moderate the association between perceived justice in tourism governance and emotional solidarity with tourists. Accordingly, we propose the following hypothesis:

***H5: Residents’ perceived tourism pressure moderates the association between perceived justice in tourism governance and residents’ emotional solidarity with tourists.***

***H5-1: Residents’ perceived tourism pressure moderates the association between distributive justice and emotional solidarity with tourists.***

***H5-2: Residents’ perceived tourism pressure moderates the association between procedural justice and emotional solidarity with tourists.***

***H5-3: Residents’ perceived tourism pressure moderates the association between interactional justice and emotional solidarity with tourists.***

To visually illustrate the hypothesized relationships among perceived justice, emotional solidarity, co-governance participation intention, and the moderating role of perceived tourism pressure, the proposed research model is presented in Fig 1.

**Fig 1:**
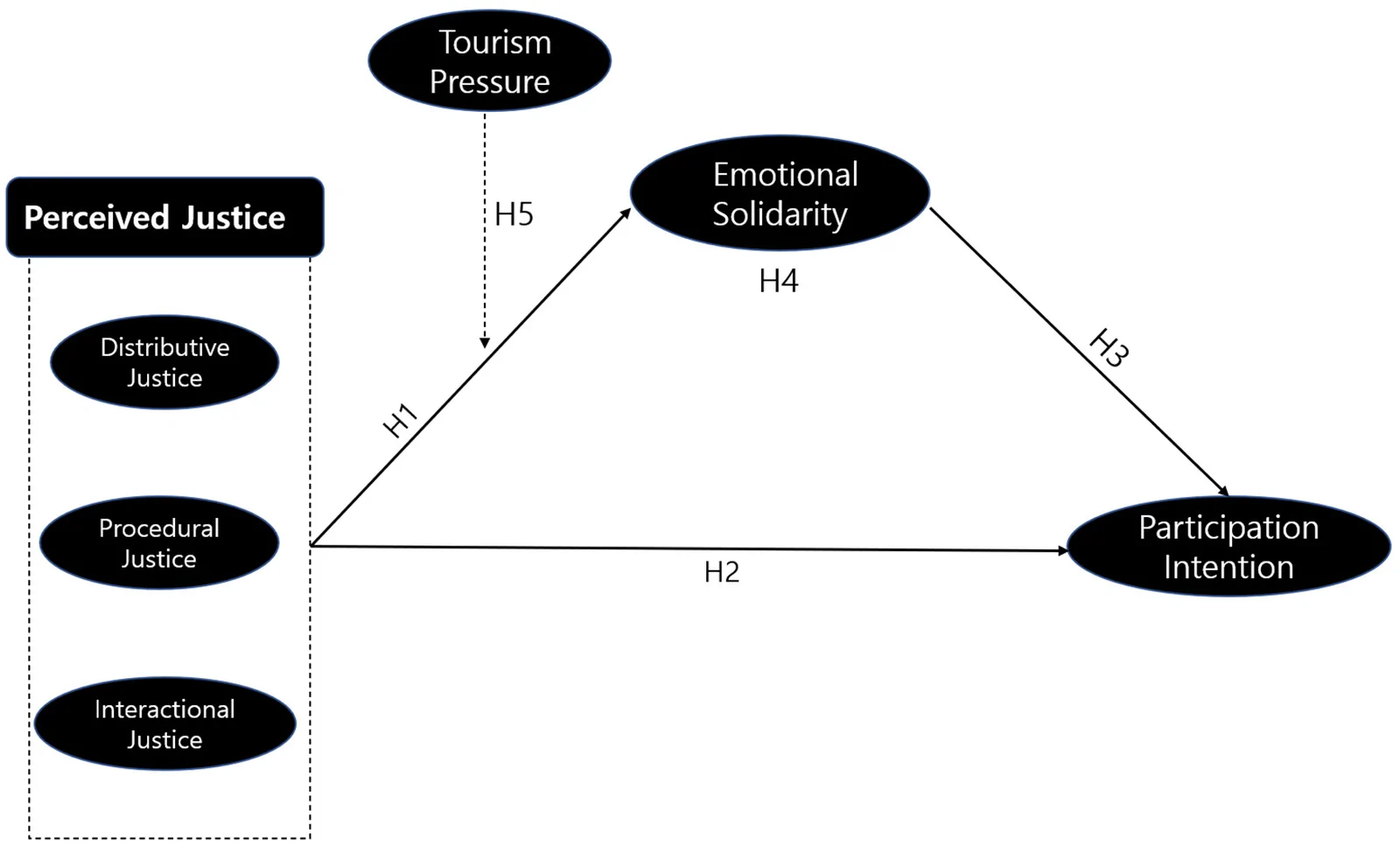
Research model and hypotheses.

## 3. Methods

### 3.1. Study context

This study was conducted in Wuxi (Jiangsu Province, China), a high-intensity urban tourism destination in the Yangtze River Delta with a permanent population of approximately 7.5 million. The city hosts four national 5A-level scenic attractions (Three Kingdoms Film and Television City, Lingshan Scenic Area, Yuantouzhu Scenic Area, and Huishan Ancient Town), and the surrounding residential communities are spatially proximate to major visitor concentrations and frequent host–guest interactions. According to the Wuxi Statistical Yearbook 2025 published by the Wuxi Municipal Bureau of Statistics, Wuxi received 146.46 million domestic tourist visits in 2024 and generated CNY 175.21 billion in tourism revenue [32]. During the 2025 Spring Festival holiday alone, the city welcomed 15.56 million visitors, indicating pronounced peak-season pressures [33]. This multi-attraction and high-flow tourism setting provides an appropriate empirical context for examining how residents’ perceived justice in tourism governance influences their emotional solidarity with tourists and their intentions to participate in tourism co-governance.

### 3.2. Measures and instrument design

Data were collected using a structured questionnaire with two sections. Section 1 measured the key constructs in the proposed model: perceived tourism governance fairness, emotional solidarity with tourists, co-governance participation intention, and perceived tourism pressure. All items were adapted from established scales and tailored to the local destination context. Tourism governance fairness was operationalized as three related but distinct first-order dimensions: distributive justice, procedural justice, and interactional justice, based on Wang et al. [22], Gautam et al. [37], and Su et al. [23]. In this study, perceived justice was treated as a conceptual umbrella for these three dimensions and was not modeled as a higher-order latent construct. Accordingly, the three justice dimensions were entered separately into the structural model. Emotional solidarity with tourists was measured with six items adapted from Woosnam and Aleshinloye [14] and Tan and Hsu [16]. Co-governance participation intention was assessed with four items adapted from Stylidis et al. [43] and Wang et al. [22]. These items captured residents’ self-reported willingness to engage in tourism-governance-related participatory processes, rather than actual or observed co-governance behavior. Perceived tourism pressure was captured using four items adapted from Jordan et al. [28,29]. All construct items were rated on a five-point Likert-type scale (1 = strongly disagree, 5 = strongly agree). Section 2 collected socio-demographic information, including gender, marital status, age, education, monthly income, occupation, and length of residence. Detailed measurement items and corresponding sources are reported in Table 1.

**Table 1:** Measurement scales and item sources.

Construct	Dimension	Item Code & Item Content	Reference
Tourism Governance Fairness	Distributive Justice	DJ1: I believe that public resources generated by tourism are fairly distributed between residents and tourists.	[22,23,37]
		DJ2: I believe that the government considers residents’ needs when providing tourism-related public services (e.g., transportation, sanitation, security).	
		DJ3: I believe that the benefits and costs of tourism (e.g., crowding, noise, rising prices) are fairly shared among different social groups.	
		DJ4: I believe that improvements gained by local communities are proportional to the intensity of tourism development.	
	Procedural Justice	PJ1: I believe that tourism planning and project decision-making processes are transparent.	
		PJ2: I believe that residents have opportunities to express their opinions during major tourism development or management adjustments.	
		PJ3: I believe that the government follows consistent and clear rules when handling tourism-related issues.	
		PJ4: I believe that effective complaint and feedback mechanisms exist when residents raise concerns about tourism governance.	
	Interactional Justice	IJ1: In tourism governance communication, I believe that government or management staff treat residents with respect.	
		IJ2: I believe that tourism governance information (e.g., policies, control measures, compensation arrangements) is explained clearly and in a timely manner.	
		IJ3: I believe that relevant authorities respond sincerely and are willing to communicate when residents raise concerns.	
		IJ4: I believe that residents are treated equally in tourism governance and are not marginalized.	
Emotional Solidarity with Tourists	Emotional Solidarity	ES1: I generally welcome tourists visiting my city or community.	[14,16]
		ES2: I am willing to interact with tourists in a friendly manner.	
		ES3: I feel emotionally willing to establish closer connections with tourists rather than remaining indifferent.	
		ES4: I believe that tourists and residents are not in opposition but can understand and get along with each other.	
		ES5: I can understand tourists’ needs and inconveniences during their travels.	
		ES6: Even when conflicts occur, I am willing to understand tourists’ behaviors from their perspective.	
Co-governance Participation Intention	Participation Intention	PI1: If the government or community organizes tourism governance consultations or hearings, I am willing to participate and express my opinions.	[22,43]
		PI2: I am willing to report tourism governance issues and offer suggestions through official platforms or community channels.	
		PI3: I am willing to participate in community-level tourism order maintenance or guidance activities (e.g., volunteering or public education).	
		PI4: Even if it requires time and effort, I am willing to participate in actions aimed at improving tourism governance.	
Perceived Tourism Pressure	Tourism Pressure	TP1: During peak tourism periods, I often feel crowded in my area, and my daily travel is clearly affected.	[28,29]
		TP2: Tourism activities make local transportation, parking, and public space usage more strained.	
		TP3: I feel that tourism development places noticeable pressure on my daily life.	
		TP4: During holidays or weekends, I deliberately avoid popular local areas to reduce inconvenience.	

As the original scales were developed in English, a back-translation procedure was applied to ensure linguistic equivalence [52]. The questionnaire was translated into Chinese and independently back-translated into English; discrepancies were resolved through consensus. A pilot test was conducted prior to the main survey to refine wording and improve clarity.

### 3.3. Sampling and data collection

Data were collected from residents living in communities surrounding four major 5A-level scenic attractions in Wuxi, namely Three Kingdoms Film and Television City, Lingshan Scenic Area, Yuantouzhu Scenic Area, and Huishan Ancient Town. A site-based intercept survey was conducted in nearby residential areas and public access points around the selected attractions over a four-week period from December 25, 2025, to January 20, 2026, covering both regular days and public holidays. Only adult residents who had lived in the surrounding communities for at least one year were invited to participate. Trained surveyors introduced the study and screened respondents for eligibility before questionnaire administration. Eligible respondents then completed a self-administered online questionnaire by scanning a QR code with their smartphones. All questionnaires were completed anonymously, and surveyors did not intervene in the response process.

A total of 382 questionnaires were collected. Before analysis, the responses were screened for eligibility and data quality. Seventeen questionnaires were excluded because missing responses prevented complete scoring of one or more study constructs. Twenty-one questionnaires were excluded because respondents selected the same response option across all or nearly all substantive Likert-scale items, indicating a clear straight-lining response pattern. Twenty questionnaires were excluded because the respondents had lived in the surrounding communities for less than one year and therefore did not meet the predefined eligibility criterion. A further 16 questionnaires were excluded because they contained logically inconsistent responses across related questions. Each excluded questionnaire was assigned to one primary exclusion category to avoid double counting. In total, 74 questionnaires were excluded, leaving a final sample of 308 valid responses for subsequent analysis. Given the site-based intercept design, the sample reflects residents living in communities surrounding the selected tourism sites rather than the general population of Wuxi.

### 3.4. Ethics statement

This study was approved by the Office of Research Administration (Science & Technology Office), Wuxi Taihu University, China, on 24 December 2025 (Ethics Review Reference No. WTH20251224A). The study involved an anonymous questionnaire survey with adult residents and was classified as a minimal-risk study in the approved ethics document. The approved ethics document specified oral confirmation and online confirmation as the informed consent procedures for offline and online data collection.

Before completing the questionnaire, all participants were informed of the study purpose, procedures, estimated time, voluntary nature of participation, anonymity, confidentiality, data use, and their right to withdraw at any time without negative consequences. For offline intercept data collection, trained surveyors provided the consent information before questionnaire administration and witnessed participants’ verbal confirmation before they proceeded with the survey. For online questionnaire completion, consent was documented through participants’ confirmation on the first page of the questionnaire. Submission of the questionnaire was considered confirmation of informed and voluntary participation.

Signed written consent was not collected because the approved consent procedure relied on oral confirmation and online confirmation, and the survey was anonymous, minimal-risk, and did not collect personally identifiable information. No names, signatures, identification numbers, telephone numbers, or other directly identifiable information were collected. All responses were de-identified and used solely for academic research.

### 3.5. Data Analysis Strategy and Statistical Procedures

All analyses were conducted in SPSS 29.0, AMOS 28.0, and PROCESS (Model 4). Before hypothesis testing, the valid sample (N = 308) was screened for univariate normality and potential common method bias. Skewness ranged from −1.592 to −0.926 and kurtosis ranged from 0.341 to 2.314, indicating acceptable departures from normality for SEM; thus, maximum likelihood estimation was applied in AMOS [53,54]. Given the cross-sectional self-report nature of the data, several procedural remedies were applied to reduce potential common method variance, including anonymous participation, confidentiality assurance, and clear instructions emphasizing that there were no right or wrong answers [55]. In addition, Harman’s single-factor test was conducted as an initial diagnostic check. The first unrotated factor explained 48.246% of the total variance, which was below the commonly used 50% threshold. However, given the limitations of Harman’s single-factor test and the proximity of this value to the threshold, this result was not considered sufficient to rule out common method variance. To provide an additional assessment, a CFA-based common method factor model was estimated by adding an unmeasured common method factor to the baseline measurement model, with equal loadings specified from the method factor to all observed indicators. The fit of this model was compared with that of the baseline CFA model to examine whether accounting for a common method factor improved model fit.

Following the two-step approach [56], a CFA was first conducted to evaluate the measurement model. Reliability and convergent validity were assessed using Cronbach’s α, CR, and AVE, while discriminant validity was examined using the Fornell–Larcker criterion and HTMT ratios [57,58]. Model fit was evaluated using CFI, TLI, RMSEA, and SRMR with conventional cutoffs [59]. Next, SEM was used to test the hypothesized associations among the study variables. Demographic variables were included as controls in the structural model, mediation analysis, and moderation analysis. These controls included gender, marital status, age, education, monthly income, residence duration, and occupation. Gender and marital status were represented by binary indicators, with male and married respondents serving as the respective reference categories. Occupation was dummy-coded into five binary variables representing student, public officer, office worker, professional, and self-employed respondents, with “Other” used as the reference category.

Indirect effects were examined using bias-corrected bootstrapping with 5,000 resamples in PROCESS Model 4 [60]. Emotional solidarity was specified as the mediator in the association between each dimension of tourism governance fairness and co-governance participation intention, with the same set of demographic controls included in each mediation model. Moderation by perceived tourism pressure was tested using a continuous interaction approach through hierarchical regression analysis. Emotional solidarity was entered as the dependent variable. In Model 1, the demographic controls, including the five occupation dummy variables, were entered. The three justice dimensions and perceived tourism pressure were entered in Model 2, followed by the three interaction terms in Model 3. The justice dimensions and perceived tourism pressure were standardized before the interaction terms were created.

For the hierarchical regression analysis, potentially influential observations among the 308 retained cases were assessed using standardized and studentized residuals, Cook’s distance, leverage values, and Mahalanobis distance. One observation had an absolute standardized residual slightly greater than 3 (standardized residual = −3.164; studentized deleted residual = −3.432), and the maximum Mahalanobis distance was 56.227. However, the maximum Cook’s distance was 0.080 and the maximum centered leverage value was 0.183. Taken together, the influence diagnostics did not indicate that any single observation exerted excessive influence on the regression estimates. Therefore, no additional cases were excluded on the basis of the outlier diagnostics.

## 4. Results

### 4.1. Demographic profile

The valid sample comprised 308 local residents. As shown in Table 2, female respondents accounted for 52.3% of the sample, while males represented 47.7%. Most participants were unmarried (61.7%). In terms of age, respondents were mainly between 21 and 50 years old (77.9%), with the largest group aged 31–40 years (29.2%). Regarding education, 33.4% held a college diploma and 29.9% had a bachelor’s degree. Monthly income was primarily concentrated between 4,001 and 8,000 yuan (36.7%), followed by 8,001–12,000 yuan (25.6%). Occupations were relatively evenly distributed, with professionals (19.2%) and office workers (18.5%) being the most common. Most respondents had lived in the study area for more than 10 years (72.7%), suggesting a high level of residential stability.

**Table 2:** Sample profile.

Category	Item	Frequency	Percentage (%)
Gender	Male	147	47.7
	Female	161	52.3
Marital status	Married	118	38.3
	Unmarried	190	61.7
Age	18-20 years	30	9.7
	21-30years	77	25
	31-40years	90	29.2
	41-50years	73	23.7
	50 years and above	38	12.3
Academic qualifications	High School and below	62	20.1
	College Diploma	103	33.4
	Bachelor’s Degree	92	29.9
	Master’s degree or above	51	16.6
Monthly Income	Below 4,000 yuan	62	20.1
	4,001–8,000 yuan	113	36.7
	8,001–12,000 yuan	79	25.6
	Above 12,001 yuan	54	17.5
Occupation	Student	52	16.9
	Public Officer	46	14.9
	Office Worker	57	18.5
	Professional	59	19.2
	Self-employed	48	15.6
	Other	46	14.9
Residence duration	1-10 Years	84	27.3
	11-15 Years	73	23.7
	16-20 Years	83	26.9
	Over 20 Years	68	22.1
Total		308	100

### 4.2. Confirmatory Factor Analysis

Confirmatory factor analysis (CFA) was conducted to assess the reliability and validity of the measurement model. The results indicated an acceptable model fit (χ^2^ = 868.925, df = 588, χ^2^/df = 1.478, IFI = 0.943, TLI = 0.936, CFI = 0.942, RMSEA = 0.040), meeting commonly recommended criteria [59].

Given the cross-sectional self-report nature of the data, common method variance was further assessed. Harman’s single-factor test was first conducted as an initial diagnostic check. The first unrotated factor explained 48.246% of the total variance, which was below the commonly used 50% threshold. However, because Harman’s single-factor test alone is limited and the value was close to the threshold, a CFA-based common method factor model was further estimated to provide an additional assessment. Specifically, an unmeasured common method factor was added to the baseline CFA model, with equal loadings specified from the method factor to all observed indicators.

As shown in Table 3, the CFA model with the common method factor also demonstrated acceptable fit (χ^2^ = 796.851, df = 587, χ^2^/df = 1.357, IFI = 0.957, TLI = 0.952, CFI = 0.957, RMSEA = 0.034). Although both the baseline CFA model and the common method factor model showed acceptable fit, the common method factor model demonstrated improved fit relative to the baseline model. This improvement indicates that some common method variance may be present and cannot be ruled out on the basis of these diagnostic tests. Accordingly, these analyses should not be interpreted as demonstrating that common method variance had no effect on the observed associations. The substantive findings are therefore interpreted with appropriate caution, given the cross-sectional and self-reported nature of the data.

**Table 3:** Comparison of CFA models for common method bias assessment.

Model	χ²	df	χ²/df	IFI	TLI	CFI	RMSEA
Baseline CFA model	868.925	588	1.478	0.943	0.936	0.942	0.04
CFA with common method factor	796.851	587	1.357	0.957	0.952	0.957	0.034

As shown in Table 4, all standardized factor loadings were significant and exceeded 0.70, ranging from 0.783 to 0.936, indicating satisfactory indicator reliability [61]. Internal consistency was supported, with Cronbach’s α values ranging from 0.929 to 0.940 and composite reliability (CR) values ranging from 0.901 to 0.956, exceeding the recommended threshold of 0.70 [57]. Convergent validity was further supported, as all average variance extracted (AVE) values ranged from 0.697 to 0.788 and exceeded the minimum criterion of 0.50.

**Table 4:** Confirmatory factor analysis.

Construct	Item Code	Estimate	AVE	CR	Cronbach’α
Distributive Justice	DJ1	0.860	0.771	0.931	0.929
	DJ2	0.865			
	DJ3	0.860			
	DJ4	0.925			
Procedural Justice	PJ1	0.862	0.788	0.937	0.935
	PJ2	0.872			
	PJ3	0.880			
	PJ4	0.936			
Interactional Justice	IJ1	0.882	0.785	0.936	0.934
	IJ2	0.867			
	IJ3	0.866			
	IJ4	0.928			
Emotional Solidarity	ES1	0.904	0.784	0.956	0.940
	ES2	0.886			
	ES3	0.846			
	ES4	0.877			
	ES5	0.934			
	ES6	0.864			
Participation Intention	PI1	0.869	0.773	0.931	0.929
	PI2	0.867			
	PI3	0.925			
	PI4	0.855			
Tourism Pressure	TP1	0.851	0.697	0.901	0.930
	TP2	0.850			
	TP3	0.783			
	TP4	0.854			

Discriminant validity was assessed using both the Fornell–Larcker criterion and the heterotrait–monotrait ratio of correlations (HTMT). As shown in Table 5, Panel A, the square roots of AVE for all constructs were greater than their corresponding inter-construct correlations, indicating adequate discriminant validity. In addition, the HTMT values ranged from 0.397 to 0.657 (Table 5, Panel B), all below the conservative threshold of 0.85 [58], providing further evidence that the constructs were empirically distinguishable. Overall, the results provide support for the reliability, convergent validity, and discriminant validity of the measurement model, supporting its suitability for subsequent structural analyses.

**Table 5:** Discriminant validity analysis.

Panel A. Fornell–Larcker criterion						
Constructs	DJ	PJ	IJ	ES	PI	TP
Distributive Justice (DJ)	0.878					
Procedural Justice (PJ)	0.421	0.888				
Interactional Justice (IJ)	0.365	0.479	0.886			
Emotional Solidarity (ES)	0.383	0.528	0.485	0.885		
Participation Intention (PI)	0.448	0.598	0.521	0.590	0.879	
Tourism Pressure (TP)	0.479	0.530	0.601	0.534	0.616	0.835
Panel B. HTMT ratios						
Construct	DJ	PJ	IJ	ES	PI	TP
DJ	—					
PJ	0.452	—				
IJ	0.397	0.495	—			
ES	0.422	0.556	0.519	—		
PI	0.475	0.619	0.54	0.623	—	
TP	0.518	0.558	0.619	0.59	0.657	—

*Note): Values on the diagonal represent the square root of the average variance extracted (AVE); off-diagonal values represent inter-construct correlations.

Notes. HTMT = heterotrait–monotrait ratio of correlations. Values below 0.85 indicate satisfactory discriminant validity. DJ = distributive justice; PJ = procedural justice; IJ = interactional justice; ES = emotional solidarity; PI = co-governance participation intention; TP = perceived tourism pressure.

### 4.3. Hypothesis testing

Structural equation modeling (SEM) was conducted to examine the hypothesized associations among the study variables while controlling for demographic characteristics. The control variables included gender, marital status, age, education, monthly income, residence duration, and occupation. Occupation was dummy-coded, with “Other” used as the reference category. The structural model demonstrated an acceptable fit to the data (χ^2^ = 1093.598, df = 474, χ^2^/df = 2.307, IFI = 0.909, TLI = 0.898, CFI = 0.908, RMSEA = 0.065). The model explained 38.8% of the variance in emotional solidarity and 51.7% of the variance in co-governance participation intention.

As reported in Table 6, distributive justice was positively associated with emotional solidarity (B = 0.163, SE = 0.070, β = 0.134, p = 0.019, 95% CI [0.026, 0.300]), supporting H1-1. Procedural justice also showed a positive association with emotional solidarity (B = 0.394, SE = 0.070, β = 0.345, p < 0.001, 95% CI [0.257, 0.531]), supporting H1-2. Interactional justice was likewise positively associated with emotional solidarity (B = 0.286, SE = 0.065, β = 0.262, p < 0.001, 95% CI [0.159, 0.413]), supporting H1-3. Among the three justice dimensions, procedural justice showed the strongest standardized association with emotional solidarity.

**Table 6:** Structural model results.

Hypothesis	Path	B	SE	β	C.R.	p	95% CI	Result
H1-1	DJ → ES	0.163	0.07	0.134	2.342	0.019	[0.026, 0.300]	Supported
H1-2	PJ → ES	0.394	0.07	0.345	5.611	<0.001	[0.257, 0.531]	Supported
H1-3	IJ → ES	0.286	0.065	0.262	4.414	<0.001	[0.159, 0.413]	Supported
H2-1	DJ → PI	0.183	0.066	0.148	2.791	0.005	[0.054, 0.312]	Supported
H2-2	PJ → PI	0.33	0.069	0.285	4.749	<0.001	[0.195, 0.465]	Supported
H2-3	IJ → PI	0.216	0.062	0.195	3.467	<0.001	[0.094, 0.338]	Supported
H3	ES → PI	0.29	0.06	0.286	4.821	<0.001	[0.172, 0.408]	Supported

**Notes.** B = unstandardized coefficient; β = standardized coefficient; SE = standard error; C.R. = critical ratio. 95% confidence intervals are reported for unstandardized estimates. DJ = distributive justice; PJ = procedural justice; IJ = interactional justice; ES = emotional solidarity; PI = co-governance participation intention. The model controlled for gender, marital status, age, education, monthly income, residence duration, and occupation. Occupation was dummy-coded, with “Other” used as the reference category.

The three justice dimensions were also positively associated with co-governance participation intention. Distributive justice was positively associated with participation intention (B = 0.183, SE = 0.066, β = 0.148, p = 0.005, 95% CI [0.054, 0.312]), supporting H2-1. Procedural justice was positively associated with participation intention (B = 0.330, SE = 0.069, β = 0.285, p < 0.001, 95% CI [0.195, 0.465]), supporting H2-2. Interactional justice was also positively associated with participation intention (B = 0.216, SE = 0.062, β = 0.195, p < 0.001, 95% CI [0.094, 0.338]), supporting H2-3. Finally, emotional solidarity was positively associated with participation intention (B = 0.290, SE = 0.060, β = 0.286, p < 0.001, 95% CI [0.172, 0.408]), supporting H3.

Overall, all hypothesized direct associations remained supported after demographic controls were included. Procedural justice showed the strongest standardized association with emotional solidarity, while emotional solidarity and procedural justice showed the strongest associations with co-governance participation intention. These results suggest that residents’ perceptions of fair procedures, respectful interactions, and emotional solidarity with tourists are closely linked to their willingness to engage in co-governance in tourism-affected communities.

### 4.4. Mediation analysis

Mediation analysis was conducted using bias-corrected bootstrapping with 5,000 resamples. Gender, marital status, age, education, monthly income, residence duration, and occupation were included as control variables. Occupation was dummy-coded, with “Other” used as the reference category. Emotional solidarity was specified as the mediator linking each dimension of tourism governance fairness to co-governance participation intention. As reported in Table 7, the bootstrap confidence intervals for all three indirect effects did not include zero, indicating significant indirect associations through emotional solidarity.

**Table 7:** Mediating effects of emotional solidarity.

Hypothesis	Mediation path	Direct effect	Indirect effect	Total effect	Proportion mediated	Boot SE	95% Bootstrap CI	Result
H4-1	DJ → ES → PI	0.258	0.1839	0.442	41.61%	0.037	[0.1197, 0.2615]	Supported
H4-2	PJ → ES → PI	0.343	0.1849	0.528	34.97%	0.036	[0.1160, 0.2598]	Supported
H4-3	IJ → ES → PI	0.281	0.1961	0.478	41.05%	0.035	[0.1292, 0.2711]	Supported

**Notes.** Indirect effects were tested using bias-corrected bootstrap procedures with 5,000 resamples, controlling for gender, marital status, age, education, monthly income, residence duration, and occupation. Occupation was dummy-coded, with “Other” used as the reference category. Total effect was obtained from the PROCESS total effect model. Proportion mediated was calculated as indirect effect divided by total effect. An indirect effect is considered significant when the 95% bootstrap confidence interval does not include zero. DJ = distributive justice; PJ = procedural justice; IJ = interactional justice; ES = emotional solidarity; PI = co-governance participation intention.

For distributive justice, the indirect effect through emotional solidarity was significant, indirect effect = 0.1839, Boot SE = 0.0368, 95% CI [0.1197, 0.2615]. The direct effect remained significant, direct effect = 0.2581, and the total effect was 0.4420. The proportion mediated was 41.61%, indicating partial mediation. For procedural justice, the indirect effect was also significant, indirect effect = 0.1849, Boot SE = 0.0358, 95% CI [0.1160, 0.2598]. The direct effect was 0.3439, the total effect was 0.5288, and the proportion mediated was 34.97%. For interactional justice, the indirect effect was significant as well, indirect effect = 0.1961, Boot SE = 0.0357, 95% CI [0.1292, 0.2711]. The direct effect was 0.2815, the total effect was 0.4777, and the proportion mediated was 41.05%.

Overall, the results support H4-1, H4-2, and H4-3. Emotional solidarity partially mediated the associations between all three dimensions of tourism governance fairness and co-governance participation intention. The proportions mediated were relatively substantial across the three mediation paths, ranging from 34.97% to 41.61%. The mediated proportion was slightly higher for distributive justice and interactional justice than for procedural justice, whereas procedural justice showed the largest total effect. These findings suggest that emotional solidarity constitutes an important relational pathway through which residents’ fairness evaluations are associated with co-governance participation intention. At the same time, the remaining significant direct effects suggest that governance fairness may also be linked to participation intention through additional mechanisms beyond emotional solidarity.

### 4.5. Moderation analysis

A hierarchical regression analysis was conducted to examine whether perceived tourism pressure moderated the associations between the three dimensions of tourism governance fairness and emotional solidarity. Emotional solidarity was entered as the dependent variable. Demographic variables were included as controls in Model 1, including gender, marital status, age, education, monthly income, residence duration, and occupation. Occupation was represented by five dummy variables, with “Other” as the reference category. Distributive justice, procedural justice, interactional justice, and perceived tourism pressure were added in Model 2, followed by the three interaction terms in Model 3. The three justice dimensions and perceived tourism pressure were standardized before the interaction terms were created. Table 8 presents the model-level statistics and coefficients for the focal predictors, while the complete regression results for all control variables, main effects, and interaction terms are provided in S1 Table.

**Table 8:** Hierarchical regression results for the continuous moderating effect of perceived tourism pressure.

Panel A. Model summary								
Model	R²	Adjusted R²	ΔR²	F change	df1		df2	p
Model 1	0.045	0.01	0.045	1.282	11		296	0.234
Model 2	0.416	0.386	0.37	46.266	4		292	<.001
Model 3	0.432	0.396	0.016	2.704	3		289	0.046
Panel B. Core regression coefficients								
Model 2								
Predictor			B	SE	β	t	p	95% CI
Distributive justice			0.063	0.043	0.079	1.473	0.142	[-.021,.146]
Procedural justice			0.214	0.044	0.27	4.849	<.001	[.127,.300]
Interactional justice			0.137	0.045	0.174	3.027	0.003	[.048,.226]
Perceived tourism pressure			0.2	0.049	0.252	4.075	<.001	[.103,.296]
Model 3								
Predictor			B	SE	β	t	p	95% CI
Distributive justice			0.049	0.046	0.062	1.07	0.286	[-.041,.138]
Procedural justice			0.217	0.046	0.275	4.723	<.001	[.127,.308]
Interactional justice			0.155	0.046	0.197	3.411	<.001	[.066,.245]
Perceived tourism pressure			0.279	0.06	0.353	4.641	<.001	[.161,.397]
Distributive justice × Perceived tourism pressure			-0.033	0.036	-0.055	-0.914	0.361	[-.103,.038]
Procedural justice × Perceived tourism pressure			0.035	0.041	0.049	0.84	0.402	[-.047,.117]
Interactional justice × Perceived tourism pressure			0.105	0.04	0.162	2.599	0.01	[.025,.184]

Note. B = unstandardized coefficient; SE = standard error; β = standardized coefficient; CI = confidence interval. Demographic controls were included in all three models. Occupation was represented by five dummy variables, with “Other” as the reference category. Justice dimensions and perceived tourism pressure were standardized before the interaction terms were created. Complete coefficients for all demographic controls and occupation dummy variables across Models 1–3 are reported in S1 Table.

As shown in Table 8, the demographic controls in Model 1 explained 4.5% of the variance in emotional solidarity, $R^{\text{2}}=\text{0}.\text{045}$, adjusted $R^{\text{2}}=\text{0}.\text{010}$. The model was not statistically significant, F(11,296)=1.282, p=0.234. After the four main predictors were added in Model 2, explained variance increased substantially to 41.6%, $R^{\text{2}}=\text{0}.\text{416}$, adjusted $R^{\text{2}}=\text{0}.\text{386}$, $\Delta R^{\text{2}}=\text{0}.\text{370}$, $F_{\text{change}}\left(\text{4},\text{292}\right)=\text{46}.\text{266}$, p<0.001. Procedural justice (β=0.270, p<0.001), interactional justice (β=0.174, p=0.003), and perceived tourism pressure (β=0.252, p<0.001) were positively associated with emotional solidarity, whereas distributive justice was not statistically significant (β=0.079, p=0.142).

In Model 3, adding the three interaction terms produced a small but statistically significant increase in explained variance, $R^{\text{2}}=\text{0}.\text{432}$, adjusted $R^{\text{2}}=\text{0}.\text{396}$, $\Delta R^{\text{2}}=\text{0}.\text{016}$, $F_{\text{change}}\left(\text{3},\text{289}\right)=\text{2}.\text{704}$, p=0.046. The interactions of distributive justice with perceived tourism pressure (B=−0.033, p=0.361, 95% CI [−0.103, 0.038]) and procedural justice with perceived tourism pressure (B=0.035, p=0.402, 95% CI [−0.047, 0.117]) were not statistically significant. By contrast, the interaction between interactional justice and perceived tourism pressure was positive and statistically significant (B=0.105, SE=0.040, β=0.162, p=0.010, 95% CI [0.025, 0.184]). Accordingly, H5 was partially supported: H5-3 was supported, whereas H5-1 and H5-2 were not supported. The maximum VIF in the final model was 2.939, and the Durbin–Watson statistic was 1.858.

The significant positive interaction for interactional justice indicates that its association with emotional solidarity became stronger as perceived tourism pressure increased. This pattern suggests that respectful communication, timely responses, and considerate treatment may be particularly salient for residents’ emotional solidarity with tourists when tourism-related pressures are perceived to be greater. In contrast, perceived tourism pressure did not significantly condition the associations of distributive justice or procedural justice with emotional solidarity. Thus, tourism pressure did not operate as a general moderator across all dimensions of governance fairness, but rather showed a selective moderating pattern for interactional justice.

### 4.6. Robustness checks

Additional robustness checks were conducted to examine the stability of the main findings. First, the structural model estimated with demographic controls was compared with the same model estimated without controls. The direction and statistical significance of the hypothesized direct paths remained unchanged after adding gender, marital status, age, education, monthly income, residence duration, and occupation as controls. This suggests that the main structural associations were not driven solely by demographic composition. Second, distributive justice, procedural justice, and interactional justice were treated as three separate first-order dimensions rather than combined into a single perceived justice score. The results reported in Tables 6 and 7 show that the main associations and indirect associations remained significant when the three justice dimensions were examined separately. Third, the mediation models were estimated separately for each justice dimension using bootstrap procedures with the same set of control variables. The indirect associations through emotional solidarity remained significant for distributive justice, procedural justice, and interactional justice, with all 95% bootstrap confidence intervals excluding zero. Taken together, these checks indicate that the main SEM and mediation findings were stable across the revised model specifications.

## 5. Discussion and conclusions

This study examined how residents’ perceived justice in tourism governance is associated with emotional solidarity with tourists and intention to participate in co-governance in a high-intensity urban destination. Integrating perceived justice theory and social exchange reasoning, the findings indicate that distributive, procedural, and interactional justice are all positively associated with residents’ socio-emotional and participatory responses. These results reinforce the view that, under conditions of sustained tourism pressure, residents evaluate tourism development not only in terms of material outcomes but also through governance legitimacy, inclusiveness, and everyday interactional experiences [1,3,19,20]. Consistent with prior tourism governance research, fair and transparent institutional arrangements appear to provide an important foundation for residents’ acceptance of tourism and their willingness to engage with destination management [22,23,27]. More importantly, the findings suggest that governance fairness may spill over into host–guest relations by being associated with how residents interpret the presence of tourists in everyday community life. When tourism governance is perceived as fair, tourists are less likely to be viewed merely as the visible source of congestion or disruption, and are more likely to be interpreted within a legitimate and manageable tourism system.

Among the three justice dimensions, procedural and interactional justice emerge as particularly salient. This suggests that residents’ emotional connection with tourists and their participatory orientation are closely associated with whether governance processes provide meaningful voice, consistent rules, and respectful communication. In high-flow destinations, where residents frequently interact with both tourists and authorities, governance is experienced as an ongoing social process rather than a set of isolated policy outcomes. The findings align with arguments that overtourism governance requires locally grounded and relationally sensitive approaches, as exclusionary or top-down practices may be associated with lower social trust and intensify perceived marginalization [6,7,36]. Distributive justice remains relevant, but the results indicate that benefit–burden allocation alone may be insufficiently associated withstrong relational and participatory outcomes in the absence of fair procedures and respectful engagement.

The mediation analysis indicates that emotional solidarity partially mediates the associations between residents’ perceived justice in tourism governance and their co-governance participation intention. Across the three justice dimensions, the mediated proportions were relatively substantial, ranging from 34.97% to 41.61%, suggesting that residents’ affective bonds with tourists may represent an important relational pathway through which fairness evaluations are linked to participatory intention. This finding is consistent with social exchange theory and prior tourism studies emphasizing the role of relational mechanisms associated with supportive behaviors [10,13,15,38]. The mediated proportion was slightly higher for distributive and interactional justice than for procedural justice, whereas procedural justice showed the largest total effect. This pattern suggests that fair procedures may be broadly associated with residents’ willingness to participate, while benefit–burden fairness and respectful interaction may rely more strongly on emotional solidarity as a relational mechanism. At the same time, the persistence of significant direct effects indicates that justice perceptions are also associated with participation intention through additional institutional pathways, such as trust in authorities and perceived governance responsiveness [21,22].

With respect to contextual conditions, the moderation analysis provides partial evidence for the role of perceived tourism pressure. Perceived tourism pressure did not significantly condition the associations of distributive or procedural justice with emotional solidarity, suggesting that residents’ evaluations of benefit–burden allocation and procedural fairness may remain relatively stable across different perceived pressure levels. However, perceived tourism pressure was significant only in the interaction between interactional justice and emotional solidarity. This pattern suggests that, in higher-pressure tourism settings, residents’ perceptions of respectful communication, timely responses, and considerate treatment become more closely linked to their affective bonds with tourists. In this sense, tourism pressure does not appear to operate as a general boundary condition for all justice dimensions. Rather, it seems to increase the salience of interactional justice in residents’ interpretation of host–guest relations under tourism-related stress [2,3].

Overall, the findings indicate that residents’ perceptions of distributive, procedural, and interactional justice are closely linked to emotional solidarity and co-governance participation intention. Procedural and interactional justice are particularly important, and emotional solidarity appears to function as a partial relational mechanism linking governance fairness to participatory intention. The moderation results further suggest that perceived tourism pressure is associated with a stronger link between interactional justice and emotional solidarity, highlighting the particular importance of respectful and responsive governance in high-pressure tourism-affected communities.

### 5.1. Theoretical implications

This study contributes to tourism governance research by conceptualizing residents’ responses to sustained tourism pressure as justice-based evaluations of governance and by showing how these evaluations are associated with host–guest relational orientations and co-governance participation intention. The findings suggest that residents’ responses are associated not only with tourism outcomes, but also by how tourism is governed and how governance is experienced in everyday life.

First, this study extends a governance-focused application of perceived justice theory by modeling distributive, procedural, and interactional justice as distinct yet complementary dimensions of tourism governance fairness. While justice theory is well established in organizational research [19–21], tourism studies have more often embedded fairness within broader assessments of impacts or exchange. By examining the three dimensions simultaneously, the findings indicate that residents evaluate tourism not only in terms of benefit–burden allocation, but also in terms of voice, rule consistency, and respectful treatment. This adds to tourism governance research by demonstrating that tourism governance fairness is a multi-dimensional institutional judgment rather than a purely outcome-based evaluation.

Second, the findings add a governance-process perspective to social exchange-based interpretations of resident responses in settings marked by sustained tourism pressure. The stronger associations of procedural and interactional justice suggest that residents are particularly sensitive to whether governance arrangements are inclusive, transparent, and communicative. This indicates that residents’ responses are associated not only with what tourism brings to the community, but also by how tourism-related decisions are made and implemented. In this sense, fair procedures and respectful interactions appear to function as important legitimacy signals that are linked to residents’ willingness to participate in co-governance.

Third, this study extends the theorization of emotional solidarity by examining it as a relational mechanism linking governance fairness to co-governance participation intention. Emotional solidarity has commonly been examined in the context of direct host–guest encounters [13,15,16], whereas the present findings suggest that it is also embedded in residents’ evaluations of tourism governance. The partial mediation results suggest that fair governance is associated with participation intention not only through institutional evaluations, but also through residents’ affective orientation toward tourists. In doing so, the study extends emotional solidarity research from general support for tourism development to a more governance-oriented form of resident engagement.

Fourth, the moderation findings refine the theoretical role of tourism pressure as a contextual condition. Rather than functioning as a general boundary condition for all dimensions of governance fairness, perceived tourism pressure was associated with a stronger link between interactional justice and emotional solidarity. This suggests that, under higher perceived tourism pressure, residents may attach greater relational significance to respectful communication, timely responses, and considerate treatment. In this way, the study adds nuance to tourism pressure research by suggesting that pressure does not simply weaken or strengthen residents’ responses uniformly; instead, it may increase the salience of everyday interactional justice in residents’ host–guest relational orientations.

Taken together, the study adds to tourism governance research by clarifying how multi-dimensional fairness evaluations are associated with residents’ emotional solidarity and co-governance participation intention in settings marked by sustained tourism pressure. It further suggests that interactional justice becomes especially salient for residents’ emotional solidarity when perceived tourism pressure is high.

### 5.2. Practical implications

From a managerial perspective, the findings suggest that residents’ co-governance participation intention under sustained tourism pressure is closely associated with whether tourism governance is perceived as procedurally fair and interactionally respectful, while emotional solidarity plays a complementary role. Because procedural and interactional justice showed stronger associations than distributive justice, practical efforts should give particular attention to the everyday governance processes through which residents experience tourism management. The moderation results further suggest that interactional justice becomes especially important when residents perceive higher levels of tourism pressure. Therefore, practical interventions should not only enhance formal governance procedures, but also emphasize respectful and responsive communication in high-pressure tourism-affected communities.

First, institutionalize procedural voice and decision transparency. Given the importance of procedural justice, local authorities should move beyond occasional consultation toward stable mechanisms that make resident participation visible and consequential. In high-pressure urban tourism settings, residents may be less concerned with abstract participation opportunities than with whether their concerns about congestion, public-space use, noise, and neighborhood disruption are heard and reflected in governance decisions. This may include regular community briefings in areas surrounding major attractions, clear standards for handling complaints related to congestion and public-space use, and publicly accessible records showing how residents’ concerns are addressed. Transparent procedures and traceable complaint-handling mechanisms can help residents see how decisions are made, how problems are prioritized, and whether local input has practical consequences. The objective is not to eliminate conflict in high-flow settings, but to reduce perceptions of arbitrariness by demonstrating consistency, explainability, and meaningful opportunities for input.

Second, strengthen interactional governance through front-line communication and service standards. Because interactional justice is closely associated with residents’ emotional and participatory responses, how policies are implemented in everyday encounters is critical. This is particularly important in communities where residents perceive stronger tourism pressure, as the results suggest that respectful communication and timely responses are more closely linked to emotional solidarity under such conditions. When tourism pressure is high, residents’ judgments of governance are often formed through immediate encounters with community workers, destination staff, security personnel, volunteers, and complaint-response channels. Destination managers can establish clear behavioral guidelines for resident-facing staff, supported by training in respectful communication, conflict mediation, and timely information delivery. Unified messaging during peak periods, rapid clarification of disruptions, and accessible feedback channels may help reduce misunderstandings and support perceptions of fair treatment. For high-pressure periods or locations, such as holidays, major events, and communities near popular attractions, authorities should prioritize rapid-response mechanisms, visible on-site coordinators, and resident-facing communication channels so that residents’ concerns are acknowledged early and consistently. These measures are especially important because respectful treatment and timely communication may make governance responsiveness more visible to residents when tourism-related pressures are most immediate.

Third, use targeted host–guest initiatives to complement fair governance. Since emotional solidarity partially links justice perceptions to participation intention, small-scale, place-based interventions can complement institutional improvements. Practical measures include on-site visitor codes of conduct, interpretation that highlights community norms, and voluntary community–visitor interaction programs near major attractions. These initiatives should aim to encourage lower social distance and mutual respect, while recognizing that they cannot substitute for fair governance arrangements. In high-pressure settings, such initiatives should be paired with concrete governance responses, such as complaint follow-up, crowd-flow coordination, and resident feedback mechanisms, because relational programs alone are unlikely to offset perceived disrespect or delayed responses.

Overall, the results indicate that residents’ co-governance participation intention is more likely to be supported when tourism management combines transparent procedures, respectful everyday interactions, and realistic relational initiatives that together are conducive to long-term trust and cooperation. Although benefit–burden allocation remains important, the stronger associations of procedural and interactional justice suggest that residents in high-pressure urban tourism communities place particular weight on being heard, informed, respected, and responded to in everyday governance processes. For destinations such as Wuxi, where major attractions generate concentrated visitor flows around surrounding communities, the practical priority is to make residents feel not only that tourism is managed fairly, but also that their everyday concerns are heard, respected, and addressed in a timely manner.

## 6. Limitations and directions for future research

This study has several limitations. First, the cross-sectional, self-reported intercept survey captures residents’ perceptions at one point in time, which limits causal inference and may inflate observed associations. Although this study applied procedural remedies, Harman’s single-factor test, and a CFA-based common method factor model to assess potential common method bias, the improved fit of the model including a common method factor indicates that some common method variance may be present and cannot be ruled out. Accordingly, the observed associations, including their magnitudes, should be interpreted with appropriate caution. In addition, because of the cross-sectional design, the findings should be interpreted as associations rather than causal relationships.

Second, the sample was drawn from communities surrounding four 5A-level attractions in Wuxi. This site-based intercept strategy was appropriate for reaching residents exposed to tourism pressure, but it was not a probability sample of the general Wuxi population. Therefore, the findings should be generalized beyond tourism-affected communities with caution. The sampling approach may also involve self-selection bias, as residents with stronger opinions about tourism development or governance issues may have been more willing to participate. In addition, the relatively young and educated sample profile, as well as the high proportion of unmarried respondents, may have shaped the observed level of willingness to participate in co-governance. Together with QR-code-based data collection, this may have underrepresented older residents, less educated residents, residents with different family responsibilities, or those less confident in using digital survey tools. Future research should adopt probability-based or stratified sampling where feasible and combine digital surveys with paper-based or interviewer-assisted methods to improve sample inclusiveness.

Third, this study measured residents’ self-reported intention to participate in tourism co-governance rather than actual co-governance behavior. Future research could examine whether such participation intention is followed by actual governance-related actions over time.

Fourth, although this study tested perceived tourism pressure using a continuous interaction approach, the analysis relied on residents’ subjective perceptions of tourism pressure. Future research should incorporate objective tourism-intensity indicators, such as visitor flows, congestion levels, or seasonal event data, to examine how perceived and objective pressure jointly relate to residents’ governance evaluations and relational responses. Future research should also use longitudinal or event-based designs across multiple cities and test additional mechanisms and boundary conditions, such as legitimacy, trust, and efficacy, using mixed methods.

## Supporting information

S1 File De-identified quantitative survey dataset. (XLS)

S2 File Survey questionnaire. (PDF)

S3 File Codebook. (PDF)

S1 Table Moderation results for perceived tourism pressure. (PDF)
