# Supplementary material for: Justice under tourism pressure: Governance fairness, emotional solidarity, and residents’ co-governance participation intention in tourism-affected communities
Source: PLoS One. 2026 Oct 12;21(10):e0360394. doi: 10.1371/journal.pone.0360394 (PMC13657307; doi:10.1371/journal.pone.0360394)
Supplement: Supplementary file 1 [file pone.0360394.s002.pdf]

Survey Questionnaire

1. Introduction and Informed Consent

Dear Participant,

This survey aims to investigate residents' perceptions and attitudes toward tourism development and tourism governance. The study is conducted solely for academic research purposes and does not involve any commercial use. This questionnaire is anonymous, and no personally identifiable information will be collected. All responses will be kept strictly confidential and used only for statistical analysis. Participation in this study is entirely voluntary, and you may withdraw at any time without any negative consequences.

The survey will take approximately 3–5 minutes to complete. Please answer all questions based on your actual feelings and experiences. There are no right or wrong answers. By proceeding with the survey, you acknowledge that you have read and understood the above information and agree to participate in this study.

2. Screening Question

S1. Are you a local resident who has lived in this area for at least one year?

□ Yes → Please continue
□ No → End of survey. Thank you for your participation

3. Main Questionnaire

The following items are designed to measure your perceptions of tourism governance fairness, emotional solidarity with tourists, co-governance participation intention, and perceived tourism pressure. Please indicate your level of agreement with each statement based on your actual experience. All items are measured on a five-point Likert scale:1 = Strongly Disagree, 5 = Strongly Agree

(1) Tourism Governance Fairness

1. Distributive Justice

Item	1	2	3	4	5
DJ1: I believe that public resources generated by tourism are fairly distributed between residents and tourists.	○	○	○	○	○

DJ2: I believe that the government considers residents' needs when providing tourism-related public services (e.g., transportation, sanitation, security).	○	○	○	○	○
DJ3: I believe that the benefits and costs of tourism (e.g., crowding, noise, rising prices) are fairly shared among different social groups.	○	○	○	○	○
DJ4: I believe that improvements gained by local communities are proportional to the intensity of tourism development.	○	○	○	○	○

2. Procedural Justice

Item	1	2	3	4	5
PJ1:I believe that tourism planning and project decision-making processes are transparent.	○	○	○	○	○
PJ2: I believe that residents have opportunities to express their opinions during major tourism development or management adjustments.	○	○	○	○	○
PJ3: I believe that the government follows consistent and clear rules when handling tourism-related issues.	○	○	○	○	○
PJ4: I believe that effective complaint and feedback mechanisms exist when residents raise concerns about tourism governance.	○	○	○	○	○

3. Interactional Justice

Item	1	2	3	4	5
IJ1: In tourism governance communication, I believe that government or management staff treat residents with respect.	○	○	○	○	○
IJ2: I believe that tourism governance information (e.g., policies, control measures, compensation arrangements) is explained clearly and in a timely manner.	○	○	○	○	○
IJ3: I believe that relevant authorities respond sincerely and are willing to communicate when residents raise concerns.	○	○	○	○	○
IJ4: I believe that residents are treated equally in tourism governance and are not marginalized.	○	○	○	○	○

(2) Emotional Solidarity with Tourists

Item	1	2	3	4	5
ES1: I generally welcome tourists visiting my city or community.	○	○	○	○	○
ES2: I am willing to interact with tourists in a friendly manner.	○	○	○	○	○
ES3: I feel emotionally willing to establish closer connections with tourists rather than remaining indifferent.	○	○	○	○	○
ES4: I believe that tourists and residents are not in opposition but can understand and get along with each other.	○	○	○	○	○

ES5: I can understand tourists' needs and inconveniences during their travels.	○	○	○	○	○
ES6: Even when conflicts occur, I am willing to understand tourists' behaviors from their perspective.	○	○	○	○	○

(3) Co-governance Participation Intention

Item	1	2	3	4	5
PI1: If the government or community organizes tourism governance consultations or hearings, I am willing to participate and express my opinions.	○	○	○	○	○
PI2: I am willing to report tourism governance issues and offer suggestions through official platforms or community channels.	○	○	○	○	○
PI3: I am willing to participate in community-level tourism order maintenance or guidance activities (e.g., volunteering or public education).	○	○	○	○	○
PI4: Even if it requires time and effort, I am willing to participate in actions aimed at improving tourism governance.	○	○	○	○	○

(4) Perceived Tourism Pressure

Item	1	2	3	4	5
TP1: During peak tourism periods, I often feel crowded in my area, and my daily travel is clearly affected.	○	○	○	○	○
TP2: Tourism activities make local transportation, parking, and public space usage more strained.	○	○	○	○	○
TP3: I feel that tourism development places noticeable pressure on my daily life.	○	○	○	○	○
TP4: During holidays or weekends, I deliberately avoid popular local areas to reduce inconvenience.	○	○	○	○	○

4. Demographic Information (Please select the option that best describes your situation):

1. Gender
□ Male □ Female

2. Marital Status
□ Married □ Unmarried

3. Age
□ 18–20 years
□ 21–30 years
□ 31–40 years

□ 41–50 years
□ 50 years and above

4. Education
□ High school or below
□ College diploma
□ Bachelor's degree
□ Master's degree or above

5. Monthly Income
□ Below 4,000 yuan
□ 4,001–8,000 yuan
□ 8,001–12,000 yuan
□ Above 12,001 yuan

6. Occupation
□ Student
□ Public officer
□ Office worker
□ Professional
□ Self-employed
□ Other

7. Residence Duration
□ 1–10 years
□ 11–15 years
□ 16–20 years
□ Over 20 years

Thank you very much for your time and participation!
