# Supplementary material for: Justice under tourism pressure: Governance fairness, emotional solidarity, and residents’ co-governance participation intention in tourism-affected communities
Source: PLoS One. 2026 Oct 12;21(10):e0360394. doi: 10.1371/journal.pone.0360394 (PMC13657307; doi:10.1371/journal.pone.0360394)
Supplement: Supplementary file 2 [file pone.0360394.s003.pdf]

Codebook for Survey Variables

1. General Description

This codebook documents the variables, measurement items, and coding procedures used in this study. The dataset is based on a structured questionnaire administered to local residents. All measurement items are reflective indicators and were assessed using a five-point Likert scale: **1 = Strongly disagree 5 = Strongly agree**. No reverse-coded items were included in this study. Data were screened prior to analysis, and incomplete or invalid responses were removed.

2. Measurement Variables

2.1 Tourism Governance Fairness (X)

(1) Distributive Justice

Variable	Item Description
DJ1	I believe that public resources generated by tourism are fairly distributed between residents and tourists.
DJ2	I believe that the government considers residents’ needs when providing tourism-related public services (e.g., transportation, sanitation, security).
DJ3	I believe that the benefits and costs of tourism (e.g., crowding, noise, rising prices) are fairly shared among different social groups.
DJ4	I believe that improvements gained by local communities are proportional to the intensity of tourism development.

(2) Procedural Justice

Variable	Item Description
PJ1	I believe that tourism planning and project decision-making processes are transparent.
PJ2	I believe that residents have opportunities to express their opinions during major tourism development or management adjustments.
PJ3	I believe that the government follows consistent and clear rules when handling tourism-related issues.
PJ4	I believe that effective complaint and feedback mechanisms exist when residents raise concerns about tourism governance.

(3) Interactional Justice

Variable	Item Description
IJ1	In tourism governance communication, I believe that government or management staff treat residents with respect.
IJ2	I believe that tourism governance information (e.g., policies, control measures, compensation arrangements) is explained clearly and in a timely manner.
IJ3	I believe that relevant authorities respond sincerely and are willing to communicate when residents raise concerns.
IJ4	I believe that residents are treated equally in tourism governance and are not marginalized.

2.2 Emotional Solidarity with Tourists (M)

Variable	Item Description
ES1	I generally welcome tourists visiting my city or community.
ES2	I am willing to interact with tourists in a friendly manner.
ES3	I feel emotionally willing to establish closer connections with tourists rather than remaining indifferent.
ES4	I believe that tourists and residents are not in opposition but can understand and get along with each other.
ES5	I can understand tourists' needs and inconveniences during their travels.
ES6	Even when conflicts occur, I am willing to understand tourists' behaviors from their perspective.

2.3 Co-governance Participation Intention (Y)

Variable	Item Description
PI1	If the government or community organizes tourism governance consultations or hearings, I am willing to participate and express my opinions.
PI2	I am willing to report tourism governance issues and offer suggestions through official platforms or community channels.
PI3	I am willing to participate in community-level tourism order maintenance or guidance activities (e.g., volunteering or public education).
PI4	Even if it requires time and effort, I am willing to participate in actions aimed at improving tourism governance.

2.4 Perceived Tourism Pressure (W)

Variable	Item Description
TP1	During peak tourism periods, I often feel crowded in my area, and my daily travel is clearly affected.
TP2	Tourism activities make local transportation, parking, and public space usage more strained.
TP3	I feel that tourism development places noticeable pressure on my daily life.
TP4	During holidays or weekends, I deliberately avoid popular local areas to reduce inconvenience.

3. Demographic Variables

**Variable**	**Description**	**Coding**
Gender	Respondent gender	1 = Male, 2 = Female
Marital status	Marital status	1 = Married, 2 = Unmarried
Education	Education level	1 = High school or below, 2 = College diploma, 3 = Bachelor’s degree, 4 = Master’s degree or above
Monthly income	Monthly income level	1 = Below 4,000 yuan, 2 = 4,001–8,000 yuan, 3 = 8,001–12,000 yuan, 4 = Above 12,001 yuan
Occupation	Occupation type	1 = Student, 2 = Public officer, 3 = Office worker, 4 = Professional, 5 = Self-employed, 6 = Other
Residence duration	Length of residence	1 = 1–10 years, 2 = 11–15 years, 3 = 16–20 years, 4 = Over 20 years
