# Supplementary material for: Justice under tourism pressure: Governance fairness, emotional solidarity, and residents’ co-governance participation intention in tourism-affected communities
Source: PLoS One. 2026 Oct 12;21(10):e0360394. doi: 10.1371/journal.pone.0360394 (PMC13657307; doi:10.1371/journal.pone.0360394)
Supplement: Supplementary file 3 [file pone.0360394.s004.pdf]

Panel A. Model-level statistics

Model	R	R^2	Adjusted R^2	SEE	ΔR^2	F change	df1	df2	p for F change	Overall F	Overall p
Model 1	0.213	0.045	0.01	0.787	0.045	1.282	11	296	0.234	1.282	0.234
Model 2	0.645	0.416	0.386	0.62	0.37	46.266	4	292	<.001	13.853	<.001
Model 3	0.657	0.432	0.396	0.614	0.016	2.704	3	289	0.046	12.197	<.001

Panel B. Model 1: Demographic controls

Predictor	B	SE	β	t	p	95% CI	VIF
Constant	4.295	0.257	—	16.74	<.001	[3.790, 4.800]	—
Female	-0.109	0.093	-0.069	-1.174	0.242	[-.293, .074]	1.079
Unmarried	0.089	0.093	0.055	0.95	0.343	[-.095, .273]	1.026
Age	0.009	0.039	0.014	0.238	0.812	[-.068, .087]	1.051
Education	-0.048	0.046	-0.06	-1.035	0.302	[-.139, .043]	1.038
Monthly income	0.011	0.045	0.014	0.24	0.81	[-.078, .100]	1.017
Residence duration	-0.017	0.041	-0.024	-0.411	0.682	[-.098, .064]	1.042
Student	-0.136	0.162	-0.065	-0.841	0.401	[-.454, .182]	1.827
Public officer	-0.103	0.166	-0.046	-0.616	0.538	[-.430, .225]	1.752
Office worker	-0.455	0.157	-0.224	-2.89	0.004	[-.765, -.145]	1.86
Professional	-0.285	0.158	-0.142	-1.797	0.073	[-.596, .027]	1.932
Self-employed	-0.36	0.164	-0.165	-2.191	0.029	[-.683, -.037]	1.768

Panel C. Model 2: Main-effects model

Predictor	B	SE	β	t	p	95% CI	VIF
Constant	4.088	0.204	—	20.087	<.001	[3.687, 4.488]	—
Female	-0.016	0.074	-0.01	-0.221	0.825	[-.162, .130]	1.1
Unmarried	0.009	0.074	0.006	0.123	0.902	[-.137, .156]	1.049
Age	0.003	0.031	0.004	0.091	0.928	[-.058, .064]	1.06
Education	-0.033	0.037	-0.042	-0.914	0.362	[-.105, .039]	1.048
Monthly income	0.019	0.036	0.024	0.524	0.601	[-.052, .089]	1.025
Residence duration	-0.009	0.033	-0.013	-0.28	0.78	[-.074, .056]	1.075
Student	-0.027	0.128	-0.013	-0.209	0.835	[-.279, .226]	1.85
Public officer	0.052	0.132	0.023	0.393	0.695	[-.207, .311]	1.769
Office worker	-0.255	0.125	-0.125	-2.037	0.043	[-.500, -.009]	1.89
Professional	-0.057	0.126	-0.028	-0.45	0.653	[-.305, .191]	1.973
Self-employed	-0.149	0.131	-0.068	-1.138	0.256	[-.406, .109]	1.806
Distributive justice	0.063	0.043	0.079	1.473	0.142	[-.021, .146]	1.447
Procedural justice	0.214	0.044	0.27	4.849	<.001	[.127, .300]	1.553
Interactional justice	0.137	0.045	0.174	3.027	0.003	[.048, .226]	1.642
Perceived tourism pressure	0.2	0.049	0.252	4.075	<.001	[.103, .296]	1.918

Panel D. Model 3: Interaction model

Predictor	B	SE	β	t	p	95% CI	VIF
Constant	3.997	0.206	—	19.433	<.001	[3.592, 4.402]	—
Female	0	0.074	0	0.002	0.998	[-.146, .146]	1.12
Unmarried	-0.014	0.075	-0.009	-0.186	0.853	[-.161, .133]	1.074
Age	0.004	0.031	0.006	0.128	0.898	[-.057, .065]	1.074
Education	-0.027	0.036	-0.034	-0.735	0.463	[-.098, .045]	1.058
Monthly income	0.021	0.036	0.026	0.575	0.566	[-.050, .091]	1.036
Residence duration	-0.009	0.033	-0.013	-0.272	0.786	[-.073, .055]	1.076
Student	-0.018	0.128	-0.009	-0.141	0.888	[-.271, .235]	1.889
Public officer	0.033	0.132	0.015	0.251	0.802	[-.226, .292]	1.794
Office worker	-0.239	0.125	-0.117	-1.906	0.058	[-.485, .008]	1.929
Professional	-0.024	0.127	-0.012	-0.186	0.853	[-.273, .226]	2.026
Self-employed	-0.139	0.13	-0.064	-1.067	0.287	[-.396, .117]	1.824
Distributive justice	0.049	0.046	0.062	1.07	0.286	[-.041, .138]	1.684
Procedural justice	0.217	0.046	0.275	4.723	<.001	[.127, .308]	1.722
Interactional justice	0.155	0.046	0.197	3.411	<.001	[.066, .245]	1.69
Perceived tourism pressure	0.279	0.06	0.353	4.641	<.001	[.161, .397]	2.939
Distributive justice × Perceived tourism pressure	-0.033	0.036	-0.055	-0.914	0.361	[-.103, .038]	1.807
Procedural justice × Perceived tourism pressure	0.035	0.041	0.049	0.84	0.402	[-.047, .117]	1.743
Interactional justice × Perceived tourism pressure	0.105	0.04	0.162	2.599	0.01	[.025, .184]	1.987

Note. B = unstandardized coefficient; SE = standard error; β = standardized coefficient; CI = confidence interval; VIF = variance inflation factor; SEE = standard error of the estimate. Female was coded with male as the reference category, and unmarried was coded with married as the reference category. Occupation was represented by five dummy variables (student, public officer, office worker, professional, and self-employed), with “Other” as the reference category. Distributive justice, procedural justice, interactional justice, and perceived tourism pressure were standardized before the interaction terms were created. The dependent variable in all models was emotional solidarity.
